# Porcine Lymphotropic Herpesvirus (PLHV) Was Not Transmitted During Transplantation of Genetically Modified Pig Hearts into Baboons

**DOI:** 10.3390/ijms26157378

**Published:** 2025-07-30

**Authors:** Hina Jhelum, Martin Bender, Bruno Reichart, Jan-Michael Abicht, Matthias Längin, Benedikt B. Kaufer, Joachim Denner

**Affiliations:** 1 Institute of Virology, Free University Berlin, 14163 Berlin, Germany; hina.jhelum@fu-berlin.de (H.J.); benedikt.kaufer@fu-berlin.de (B.B.K.); 2 Department of Anaesthesiology, University Hospital, Ludwig-Maximilians-University Munich, 81377 Munich, Germany; martin.bender@med.uni-muenchen.de (M.B.); jan.abicht@med.uni-muenchen.de (J.-M.A.); matthias.laengin@med.uni-muenchen.de (M.L.); 3 Walter Brendel Centre, Ludwig-Maximilians-University Munich, 81377 Munich, Germany; bruno.reichart@med.uni-muenchen.de

**Keywords:** porcine lymphotropic herpesviruses (PLHV), xenotransplantation, pig, baboon, virus safety, PCR, porcine cytomegalovirus/porcine roseolovirus (PCMV/PRV)

## Abstract

Porcine lymphotropic herpesviruses -1, -2, and -3 (PLHV-1, PLHV-2, and PLHV-3) are gammaherpesviruses that are widespread in pigs. These viruses are closely related to the human pathogens Epstein–Barr virus (EBV) and Kaposi sarcoma-associated herpesvirus (KSHV), both of which are known to cause severe diseases in humans. To date, however, no definitive association has been established between PLHVs and any disease in pigs. With the growing interest in xenotransplantation as a means to address the shortage of human organs for transplantation, the safety of using pig-derived cells, tissues, and organs is under intense investigation. In preclinical trials involving pig-to-nonhuman primate xenotransplantation, another porcine herpesvirus—porcine cytomegalovirus, a porcine roseolovirus (PCMV/PRV)—was shown to be transmissible and significantly reduced the survival time of the xenotransplants. In the present study, we examined donor pigs and their respective baboon recipients, all of which were part of preclinical pig heart xenotransplantation studies, for the presence of PLHV. PLHV-1, PLHV-2, and PLHV-3 were detected in nearly all donor pigs; however, no evidence of PLHV transmission to the baboon recipients was observed.

## 1. Introduction

Xenotransplantation using cells or organs from genetically modified pigs is progressing toward clinical application. Following numerous successful preclinical trials involving the transplantation of pig organs into non-human primates, the first transplantations of pig hearts and kidneys into human patients have been performed [1]. In addition to challenges such as immune rejection and physiological incompatibility, the risk of transmitting porcine microorganisms remains a significant hurdle [2]. The transmission of porcine cytomegalovirus, a porcine roseolovirus (PCMV/PRV), to the first human recipient of a pig heart [3] highlighted the need for highly sensitive methods to detect porcine viruses in donor pigs and effective strategies to prevent such transmissions.

Porcine lymphotropic herpesviruses -1, -2, and -3 (PLHV-1, PLHV-2, and PLHV-3), also called suid gammaherpesviruses 3, 4, and 5 (SuHV-3, SuHV-4, and SuHV-5), are viral species of the *Macavirus* genus, *Gammaherpesvirinae* subfamily, within the *Herpesviridae* family. SuHV-3/PLHV-1 and SuHV-4/PLHV-2 were first reported by Ehlers et al. in 1999 [4]. Later, another suid gammaherpesvirus was found with considerable genomic differences relative to PLHV-1 and PLHV-2 and named suid gammaherpesvirus 5 (porcine lymphotropic herpesvirus 3—PLHV-3) [5]. For the sake of simplicity, we will stick to the designations PLHV-1, PLHV-2, and PLHV-3.

Under natural conditions there were no reports that PLHV-1, PLHV-2, and PLHV-3 represent primary pathogens of pigs or co-factors in other viral infections [6]. Despite their high prevalence, PLHVs’ relevance for the pig industry appears low. Their transmission occurs mainly horizontally, but vertical transmission is possible [7,8].

PLHV-1 has been associated with a form of post-transplantation lymphoproliferative disorder (PTLD) in immunosuppressed pigs undergoing experimental allogenic bone marrow transplantations [9,10,11]. The clinical symptoms of experimental porcine PTLD, such as fever, lethargy, anorexia, high white blood cell count, and palpable lymph nodes, are similar to those of human PTLD, a serious complication of solid human organ and allogeneic bone marrow transplantation linked to Epstein–Barr virus (EBV) (human herpesvirus-4, HHV-4) [11].

In studies published to date analyzing the prevalence of PLHV [12,13,14,15,16,17,18,19,20,21,22,23,24,25,26] (Table 1), both PCR-based methods and immunological assays have been employed. The publications were selected based on their screening for PLHV or reporting on PLHV prevalence. The PCR approaches used primers and probes targeting sequences of the DNA polymerase and glycoprotein B genes. Immunological methods, including Western blot and ELISA, were used to detect antibodies against PLHV as indirect indicators of infection. For example, ELISA-based testing revealed seropositivity rates ranging from 38% in piglets to 90% in gilts and 100% in breeding sows and pigs intended for slaughter [27]. The presence of high antibody titers in newborn piglets, which decline over time, indicates the transfer of virus-specific antibodies via colostrum from infected mother sows [27]. This pattern has also been observed in cases involving PCMV/PRV [28].

PLHVs are closely related to alcelaphine herpesvirus type 1, AlHV-1, and ovine herpesvirus type 2, OvHV-2, two gammaherpesviruses, which are apathogenic in their natural hosts but cause serious lymphoproliferative diseases in other species [29]. Accordingly, PLHV may be apathogenic in pigs but pathogenic in other species, including humans. Here, we demonstrate that although PLHV-1, PLHV-2, and PLHV-3 were present in the donor pigs, they were not transmitted to the baboon recipient of the pig heart.

## 2. Results

### 2.1. Presence of PLHV in Donor Pigs for Heart Xenotransplantation

Thirteen donor pigs and the corresponding baboon recipients were tested for PLHV-1, PLHV-2, and PLHV-3. The immunosuppressive regimen was based on a co-stimulation blockade targeting the CD40/CD40 L pathway, as previously described in detail [30]. For induction therapy, animals additionally received a B cell–depleting monoclonal antibody and a T cell–directed immunomodulatory agent. Maintenance therapy consisted of continued co-stimulation blockade in combination with an antiproliferative compound and corticosteroids. PCMV/PRV from the donor pig was transmitted to three of the 10 baboons. The presence of the porcine cytomegalovirus, which is actually a roseolovirus (PCMV/PRV) [31] (in some pigs and baboons at very high virus loads) contributed to a short survival time of these transplants [32] (Table 2). Whereas only three donor pigs were PCMV/PRV positive, all pigs were tested positive for PLHV-1 or -2, but none of them were positive for PLHV-3. To test for PLHV-1, PLHV-2, and PLHV-3, two conventional PCR methods, one detecting PLHV-1 and PHLV-2 and the other PHLV-3, were performed. Although PLHV-1 or PHLV-2 were found in all donor pigs, no virus was found in the recipient baboons (Table 2). The following baboon tissues were analyzed: spleen, liver, lung, and kidney. None of these tissues tested positive for PLHV-1 or PLHV-2.

### 2.2. Real-Time PCR-Based Detection of PLHV in Donor Pigs

To investigate the situation in greater detail, real-time PCR assays were developed and employed to detect PLHV-1, PLHV-2, and PLHV-3. Samples were collected from the liver and spleen of three new donor pigs (7649, 7654, and 7687), as well as from tissues of the corresponding baboon recipients (A, B, and C). These animals had survival times over 150 days, with the exception of baboon B, with only one day. The tissues analyzed included the liver and spleen of the baboons—except for baboon B, for which no tissues were available—and the left and right ventricles of the explanted pig hearts retrieved post-mortem (Table 3). Importantly, one donor pig, animal 7649, tested negative for all three PLHV viruses. This pig was the only one among the 11 animals tested to be completely free of PLHV. In contrast, pig 7654 was positive for PLHV-2 in both the liver and spleen. Pig 7687 was found to be co-infected with PLHV-1 and PLHV-3, with both viruses present in both organs.

None of the PLHV viruses were detected in any of the transplanted baboons or in the explanted pig hearts. This indicates that PLHV-1, PLHV-2, and PLHV-3 were not transmitted to the baboons. Although the presence of PLHV might be expected in the transplanted pig hearts—given the viral detection in the donor liver and spleen—the viral load was probably below the detection threshold of our assay.

The detection limit of the real-time PCR method used was 1 copy per 100 ng of total DNA for all three PLHV targets [18]. PCMV/PRV was not detected in any of the donor pigs, the transplanted baboons, or the explanted pig hearts.

To assess the presence of pig cells in baboon tissues (microchimerism), we performed a Short Interspersed Nuclear Elements (SINE) PCR and detected pig-specific sequences in all baboon tissue samples (Table 3). This indicates that pig cells were present in all analyzed baboon organs. These findings are consistent with previous results obtained using the same SINE PCR assay [34], as well as in a case of transplantation of a PCMV/PRV-positive heart, with immunohistochemical analyses employing a PCMV/PRV-specific antibody [35]. The high load of SINE sequences in the right and left ventricles is not surprising, as the tissue is of pig origin. Surprisingly, the ventricles explanted from baboon A show a particularly low load of SINE sequences—levels even lower than those observed in the baboon’s liver and spleen. This discrepancy suggests that incorrect material may have been submitted for testing.

However, if any of the pig cells present in the baboon tissues were positive for PLHV, their number was likely below the detection threshold of our assay, and thus the viruses could not be detected.

The results clearly indicate that all three PLHV, PHLV-1, PHLV-2, and PHLV-3, were not replicating in the transplanted pig heart and were not infecting baboon cells.

## 3. Discussion

We demonstrated that although PLHV-1, PLHV-2, and PLHV-3 were present in the donor pigs, these viruses were not transmitted to the non-human primate recipients of the pig hearts. This contrasts sharply with the case of PCMV/PRV, which was consistently transmitted to recipients regardless of the species (baboon or cynomolgus monkey), the transplanted organ (kidney or heart), or the transplantation method (heterotopic or orthotopic) [32,36,37,38]. Although a Western blot assay which we had established at the Robert Koch Institute in Berlin to screen for antibodies against PLHV [13], was not available, it is unlikely that this assay would have produced a different result. PCMV/PRV transmission significantly reduced graft survival. Notably, in orthotopic heart transplantations, survival time dropped from 195 days to fewer than 20 days [32]. PCMV/PRV was identified as the cause of multiorgan failure, altered cytokine profiles, and coagulation abnormalities [32]. The virus was also transmitted to the first human patient who received a pig heart transplant in Baltimore, where it likely contributed to the patient’s death [3,39]. Importantly, PCMV/PRV transmission occurred even when the virus was undetectable in the donor pig by PCR due to its latent state—this was observed both in a baboon case [40] and in the first human xenotransplantation patient [3,39]. Although PCMV/PRV does not infect human or non-human primate cells in vitro and does not affect healthy humans in contact with pigs or consuming undercooked pork, it causes disease specifically in the context of xenotransplantation [41].

When Mueller et al. [21] conducted transplantations of thymokidneys, kidneys, and hearts from Large White/Landrace cross-breed pigs transgenic for human decay-accelerating factor, as well as from Massachusetts General Hospital (MGH) miniature swine, they found that 78% of the donor pigs were positive for PLHV-1 and 25% for PLHV-2. All donor animals tested positive for PCMV/PRV. In the recipient baboons, the authors observed strong replication of PCMV/PRV in the explanted pig organs. However, no increase in PLHV-1 viral load was detected in xenografts that were PLHV-1–positive. PLHV was primarily localized to lymphoid tissues. Despite immunosuppression and the presence of xenogeneic immune responses, neither PLHV-1–positive xenotransplants nor those negative at baseline showed signs of PLHV-1 activation. The authors suggested that the lack of PLHV replication may be due to the absence of a sufficient number of target cells capable of supporting PLHV-1 replication within solid-organ xenografts.

When Issa et al. [42] transplanted organs from pigs transgenic for human decay-accelerating factor or from alpha-1,3-galactosyltransferase gene-knockout miniature pigs into baboons, PLHV-1 DNA was detected in peripheral blood mononuclear cells (PBMCs) of 6 out of 10 transplanted baboons. However, there was no evidence of productive PLHV-1 infection, as viral loads in the serum did not increase over time, even with prolonged transplant survival. The authors concluded that the presence of viral DNA likely resulted from persistent pig cell microchimerism rather than active viral replication. While PCMV/PRV can be effectively eliminated from source animals through early weaning of piglets [43,44], this strategy failed to exclude PLHV-1 [43]. This difference underscores the distinct mechanisms of infection and latency between PCMV/PRV and PLHV-1, two unrelated herpesviruses.

In our first experiment, summarized in Table 2, PLHV-3 was not detected in any of the donor pigs, PLHV-1 was detected in eight and PLHV-2 in two pigs. In contrast, in the second experiment (Table 3), all three PLHV types were detected. Notably, this experiment marked the first time a PLHV-free animal was identified in our facility, as well as the first documented case of co-infection with PLHV-1 and PLHV-3 (Table 3).

Co-infections with two or even all three PLHV types are not uncommon. In a screening of 21 indigenous Greek black pigs, six animals were infected with all three viruses, and the remaining animals were infected with two [18]. Among them, six pigs carried PLHV-1 and PLHV-3, while nine were infected with PLHV-2 and PLHV-3 [18]. In contrast, all 10 German slaughterhouse pigs tested were positive for PLHV-1 and PLHV-3, but not for PLHV-2 [20]—the same combination found in animal 7687 (Table 3).

The absence of PLHV in the explanted pig hearts after prolonged survival times of more than 150 days indicates that the virus present in the donor pig was not replicating in the xenotransplant, even under conditions of immunosuppression and extended survival.

Regarding the genetic relatedness of the three PLHV viruses, PLHV-3 is significantly more distantly related to PLHV-1 and PLHV-2 than PLHV-1 and PLHV-2 are to each other [5].

As mentioned in the introduction, the gene content of porcine lymphotropic herpesviruses (PLHVs) is highly similar to that of alcelaphine herpesvirus 1 (AlHV-1), associated with wildebeests, and ovine herpesvirus 2 (OvHV-2), associated with sheep [5,26,45]. These viruses cause malignant catarrhal fever (MCF), a lymphoproliferative and inflammatory disease that is typically fatal in susceptible species. PLHVs are also related to bovine lymphotropic herpesvirus (BLHV), which is associated with bovine leukemia [46], and caprine herpesvirus 2 (CprHV-2), which has been linked to chronic disease in sika deer [47,48]. While BLHV is believed to contribute to the pathogenesis of bovine leukemia virus (BLV) infection [46], and CprHV-2 is implicated in chronic illness in cervids [49], AlHV-1 and OvHV-2 are well adapted to their natural hosts—wildebeests and sheep, respectively—and remain apathogenic in those species. However, when transmitted to non-natural hosts such as cattle or deer, they can cause MCF [29]. Therefore, the potential for PLHVs to cause disease in xenotransplantation recipients cannot be excluded.

PLHV-1, -2, and -3 are also related to the human gammaherpesviruses Epstein–Barr Virus (EBV or human herpesvirus 4, HHV-4) and the human Kaposi sarcoma-associated herpesvirus (KSHV, or human herpesvirus 8, HHV-8). Moreover, they are related to EBV and KSHV in terms of B cell tropism, and sequence similarity of conserved genes [4,5,50]. Although PLHV, HHV-4 and HHV-8 belong to the *Gammaherpesvirinae* subfamily, they fall into different genera within that subfamily. PLHV are macaviruses, HHV-4 is a lymphocryptovirus, and HHV-8 is a rhadinovirus. PLHV-1 is more closely related to the rhadinoviruses (the genus that includes HHV-8) than to lymphocryptoviruses like HHV-4. The sequence homology is limited and mostly confined to conserved core genes (e.g., DNA polymerase, terminase, and capsid proteins).

PLHV-3 was found in the permanent porcine B cell line L23 and two other lymphoma cell lines, 1B2 and 2F8 [5,33]. EBV is associated with a post-transplantation lymphoproliferative disorder (PTLD) occurring in the setting of iatrogenic immune suppression following hematopoietic or solid organ transplant [51,52]. A similar disorder was observed in PLHV-1-positive minipigs after experimental allogenic bone marrow transplantations [12,13,14]. In the genome of PLHV-1, three genes encoding for putative immediate-early gene (IE) transactivators (ORF50, ORFA6/BZLF1 h, and ORF57) were found which are homologous to transactivators of HHV-8 and EBV [53], demonstrating the relationship of these viruses.

Several herpesviruses contain open reading frames that encode viral G protein-coupled receptors (vGPCRs), which they acquired from the host. BILF1 is the vGPCR encoded by EBV; it is a molecule with immunoevasive properties associated with MHC-I cell surface downregulation [54,55,56], thereby preventing recognition of EBV-infected cells by CD8+ T cells [57]. BILF1 has not only an immunoevasive properties, but also acts as an oncogene [58,59]. The BILF1 orthologues from PLHV1–3 are similar to EBV-BILF1 regarding cell surface localization, constitutive internalization, and ability to downregulate MHC-I [60]. PLHV1-BILF1 was found upregulated in lymphatic tissue from diseased miniature pigs with PTLD [60]. This data suggests that like EBV-BILF1, PLHV1–3-BILFs could have cell-transforming properties.

Given these findings and given that PLHV-1 transactivators are capable of upregulating EBV and HHV-8 promoters [61], it is advisable to use PLHV-free donor pigs for xenotransplantation. However, eliminating PLHV-1 to -3 from donor herds poses significant challenges. Early weaning has proven ineffective [43], there are currently no antiviral treatments or vaccines available, and although cross-placental transmission of PLHV is extremely rare, it remains possible [11]. Nonetheless, as demonstrated in this study, PLHV-negative animals—such as pig 7649—do exist and can be selectively bred for use in xenotransplantation.

## 4. Materials and Methods

### 4.1. Animals and Tissue Samples

Pigs 5528, 5415, 5420, 5623, 5803, 5807, and 6249, all of which were triple genetically modified, served as heart donors for baboons J, K, L, M, O, N, P, and Q, as previously described [30]. Pigs 6827 and 7094 served as donors for baboons X and Y, respectively (Table 2). Donor pigs were screened using blood samples, while formalin-fixed tissues were used for testing the baboon recipients [32].

Similarly, pigs 7649, 7654, and 7687, which were also triple genetically modified (α1,3-galactosyltransferase-knockout and expressing human CD46 and thrombomodulin), were used as heart donors for baboons A, B, and C. Orthotopic pig-to-baboon cardiac xenotransplantation using continuous non-ischemic preservation and pharmacological growth inhibition was performed as previously described in detail [30]. The immunosuppressive regimen was based on a co-stimulation blockade targeting the CD40/CD40 L pathway, as previously detailed [30]. For induction therapy, animals additionally received a B cell–depleting monoclonal antibody and a T cell–directed immunomodulatory agent. Maintenance therapy consisted of continued co-stimulation blockade in combination with an antiproliferative compound and corticosteroids.

### 4.2. Blood Collection

Blood sampling from adult sows was performed without sedation under manual fixation. Whole blood was drawn from the jugular vein with single-use needles (Ehrhardt Medizinprodukte, Geislingen, Germany) into lithium heparin and serum Monovettes (Sarstedt, Nümbrecht, Germany).

### 4.3. DNA Isolation

Formalin-fixed tissues from baboons J, K, L, M, O, N, P, and Q were cut into small pieces, and DNA was isolated according to the manufacturer’s instructions using the QIAamp DNA FFPE Tissue Kit (QIAGEN, Hilden, Germany) [32].

DNA was isolated from frozen tissue samples according to the manufacters’ instructions using the DNeasy Blood and Tissue Kit (QIAGEN, Hilden, Germany). DNA concentrations were determined using a NanoDrop ND-1000 (Thermo Fisher Scientific Inc., Worcester, MA, USA).

### 4.4. PCR and Real-Time PCR

Pigs and baboons listed in Table 2 were tested using a conventional PCR detecting PLHV-1 and PLHV-2 (PLHV-1 gives a 400 bp band, PLHV-2 gives a 343 bp band) and a conventional PCR detecting PLHV-3 (Table 4) as described [5,32,62]. PCMV/PRV was screened using a real-time PCR [32]. Animals listed in Table 3 were tested using real-time PCRs with specific primers and probes. The sensitivity to detect PCMV/PRV (sensitivity 10 copies/100 ng DNA), PLHV-1 (1 copy/100 ng DNA), PLHV-2 (1 copy/100 ng DNA), and PLHV-3 (1 copy/100 ng DNA) was described previously [18]. The primers and probes are listed in Table 4. All protocols were performed using the SensiFAST Probe No-ROX Kit (Meridian Bioscience, Cincinnati, OH, USA) in a reaction volume of 16 μL plus 4 μL (100 ng) of DNA template. Duplex real-time PCRs were performed, testing simultaneously the viral gene of interest and porcine/human glyceraldehyde-3-phosphate-dehydrogenase (p/hGAPDH) as an internal control. The functionality of the PCRs was verified using virus-specific gene blocks containing the sequence of the primers and the probe [17]. Real-time PCR reactions were carried out using a qTOWER3 G qPCR cycler (Analytik Jena, Jena, Germany) and the real-time PCR conditions as previously described [18].

## 5. Conclusions

PLHV-1, PLHV-2, and PLHV-3 are widely distributed among pigs, including those bred specifically for xenotransplantation. These viruses can be readily detected using PCR-based methods. Although PLHV-1, PLHV-2, and PLHV-3 have been identified in donor pigs, no transmission to transplanted baboons has been observed to date using PCR-based methods. Nevertheless, it is recommended to use virus-free animals, as some related gammaherpesviruses have been shown to cause severe disease following trans-species transmission.

## Figures and Tables

**Table 1:** Prevalence of PLHV-1, -2, and -3 in pigs.

Pig Breed	Detection of Virus Using Real-Time PCR			Western Blot Analysis ^b^	Reference
	PLHV-1 ^a^	PLHV-2 ^a^	PLHV-3 ^a^		
	Number of Positive Animals/Number of Tested Animals				
Göttingen minipigs 1	0/10 (0%)	0/10 (0%)	0/10 (0%)	1/10 (0%)	Morozov et al., 2016 [12]
Göttingen minipigs 2	0/10 (0%)	0/10 (0%)	0/10 (0%)	0/10 (0%)	Plotzki et al., 2016 [13]
Göttingen minipigs 3	2/11 (18%)	2/11 (18%)	2/11 (18%)	n.t. ^c^	Krüger et al., 2019 [14]
Göttingen minipigs with DPS ^d^	0/7 (0%)	0/7 (0%)	1/4 (25%)	n.t.	Jhelum et al., 2023 [15]
Aachen minipigs	0/18 (0%)	5/18 (28%)	2/18 (16%)	0/10 (0%)	Plotzki et al., 2016 [16]
Mini LEWE	0/10 (0%)	0/10 (0%)	0/10 (0%)	n.t.	Halecker et al., 2021 [17]
Indigenous Greek black pigs	12/21 (57%)	15/21 (71%)	21/21 (100%)	n.t.	Jhelum et al., 2024 [18]
Greek pigs with erythema multiforme	5/5 (100%)	1/5 (20%)	4/5 (80%)	n.t.	Halecker et al., 2022 [19]
German slaughterhouse pigs 1	2/36 (6%)	0/36 (0%)	10/36 (28%)	7/36 (19%)	Plotzki et al., 2016 [13]
German slaughterhouse pigs 2	10/10 (100%)	0/10 (0%)	10/10 (100%)	n.t.	Jhelum et al., 2024 [20]
Large White × Landrace	12/22 (55%)	2/22 (9%)	n.t.	n.t.	Mueller et al., 2004 [21]
Farm animals, Poland	11/45 (24%)	n.t.	n.t.	n.t.	Cybulski et al. 2025 [22]
Farm animals, Brazil	12/19 (63%)	n.t.	n.t.	n.t.	Dall Agnol et al., 2020 [23]
Italien pigs	50/168 (30%)	19/168 (11%)	7/168 (7%)	n.t.	Franzo et al., 2021 [24]
German pigs, lung spleen Italien pigs, blood	21/27 (78%) 20/34 (59%) 16/20 (80%)	11/27 (41% 9/34 (26%) 4/29 (20%)	16/27 (59%) 21/34 (62%) 13/20 (65%)	n.t.	Chmielewicz et al., 2003 [5]
Farm pigs, Ireland	25/32 (78%)	7/32 (22%)	37/65 (60%)	n.t.	McMahon et al., 2006 [25]
Wild boars, Brazil	48/50 (96%)	28/50 (56%)	22/50 (44%)	n.t.	Porto et al., 2021 [26]

^a^ PLHV-1, PHLV-2, PHLV-3, porcine lymphotropic herpesviruses -1, -2, -3; ^b^ using the Western blot analysis, antibodies against PLHV were detected; ^c^ n.t., not tested; ^d^ dippity pig syndrome.

**Table 2:** Testing donor pigs and recipient baboons for PLHV-1, -2, -3, and PCMV/PRV.

Animal	ID Number	Transplant Survival Days	PCMV/PRV Real-Time PCR (ct)	PLHV-1 PCR	PLHV-2 PCR	PLHV-3 PCR
Pig 5528			n.d.	Pos.	Neg.	Neg.
Baboon J	17,186	90	n.d.	Neg.	Neg.	Neg.
Pig 5415			n.d.	Pos.	Neg.	Neg.
Baboon K	17,187	50	n.d.	Neg.	Neg.	Neg.
Pig 5420			n.d.	Pos.	Neg.	Neg.
Baboon L	17,290	90	n.d.	Neg.	Neg.	Neg.
Pig 5623			27.	Pos.	Neg.	Neg.
Baboon M	17,188	10	26	Neg.	Neg.	Neg.
Pig 5803			n.d.	Pos.	Neg.	Neg.
Baboon O	17,493	195	n.d.	Neg.	Neg.	Neg.
Pig 5807			n.d.	Pos.	Neg.	Neg.
Baboon N	17,491	182	n.d.	Neg.	Neg.	Neg.
Pig 6249			29	Pos.	Neg.	Neg.
Baboon P	17,494	15	28	Neg.	Neg.	Neg.
Pig 6253			30	Pos.	Neg.	Neg.
Baboon Q	17,492	27	32	Neg.	Neg.	Neg.
Pig 6827			n.d.	Neg.	Pos.	Neg.
Baboon X		28	n.d.	Neg.	Neg.	Neg.
Pig 7094			n.d.	Neg.	Pos.	Neg.
Baboon Y		90	n.d,	Neg.	Neg.	Neg.
L23 cells				Neg.	Neg.	Pos.

ID number, identification number; PCMV/PRV, porcine cytomegalovirus/porcine roseolovirus; PLHV-1, PLHV-2, and PLHV-3, porcine lymphotropic herpesviruses -1, -2, and -3; n.d., not detected, negative real-time PCR result; Neg., negative result of the conventional PCR; Pos., positive result of the conventional PCR; L23 cells, porcine B cell line, positive for PLHV-3 [5,33]. The following baboon tissues were tested: spleen, liver, lung, and kidney.

**Table 3:** Screening of donor pigs and transplanted baboons for PLHV-1, PLHV-2, and PLHV-3 using real-time PCRs.

Animal	Organ Tested	PCMV/PRV Real-Time PCR ct	PLHV-1		PLHV-2		PLHV-3		SINE	
			Real-Time PCR ct	Copy Number	Real-Time PCR ct	Copy Number	Real-Time PCR ct	Copy Number	Real-Time PCR ct	Copy Number
Pig 7649	Spleen	n.d.	n.d		n.d		n.d		n.t.	
	Liver	n.d	n.d		n.d		n.d		n.t.	
Baboon A	Spleen	n.d	n.d		n.d		n.d		16.86	10^6^
	Liver	n.d	n.d		n.d		n.d		23.12	10^4^
	RV ^a^	n.d	n.d		n.d		n.d		28.48	10^3^
	LV ^b^	n.d	n.d		n.d		n.d		28.00	10^3^
Pig 7654	Spleen	n.d	n.d		26.38	10^3^	n.d		n.t.	
	Liver	n.d	n.d		26.94	10^3^	n.d		n.t.	
Baboon B	RV	n.d	n.d		n.d		n.d		7.05	10^10^
	LV	n.d	n.d		n.d.		n.d		7.06	10^10^
Pig 7687	Spleen	n.d	27.51	10^3^	n.d		35.13	10^1^	n.t.	
	Liver	n.d	28.51	10^2^	n.d		35.17	10^1^	n.t.	
Baboon C	Spleen	n.d	n.d		n.d		n.d		19.55	10^6^
	Liver	n.d	n.d		n.d		n.d		20.64	10^5^
	RV	n.d	n.d		n.d		n.d		7.2	10^10^
	LV	n.d	n.d		n.d		n.d		6.54	10^10^

^a^ right ventricle of the explanted pig heart; ^b^ left ventricle of the explanted pig heart; n.d., not detected by PCR; n.t., not tested.

**Table 4:** Primers and probes.

Primers Used for PCR	Sequence 5′-3′	Nucleotide Position	Accession Number	Reference
Primers used for PCR				
PLHV-1,-2 (747) fw PLHV-1,-2 (747) rev	CAYGGTAGTATTTATTCAGACA GATATCCTGGTACATTGGAAAG	21,146–21,167 21,488–21,467	AF478169.1	Ehlers B., 2002 [62]
PLHV-3 (905) fw PLHV-3 (905) rev	ACAAGAGCCTTAGGGTTCCAAACT GTGTCCAGTGTTGTAATGGATGCC	13,472–13,495 13,727–13,704	AY170316.1	Chmielewicz et al., 2003 [5]
Primers and probes used for real-time PCR				
pGAPDH fw pGAPDH rev pGAPDH probe	ACATGGCCTCCAAGGAGTAAGA GATCGAGTTGGGGCTGTGACT HEX-CCACCAACCCCAGCAAGAGCACGC-BHQ1	1,083–1,104 1,188–1,168 1,114–1,137	NM_001206359.1	Duvigneau et al., 2005 [63]
hGAPDH fw hGAPDH rev hGAPDH probe	GGCGATGCTGGCGCTGAGTAC TGGTTCACACCCATGACGA HEX-CTTCACCACCATGGAGAAGGCTGGG-BHQ1	3,568–3,587 3,803–3,783 3,655–3,678	AF261085	Behrendt et al., 2009 [64]
PCMV/PRV fw PCMV/PRV rev PCMV/PRV probe	ACTTCGTCGCAGCTCATCTGA GTTCTGGGATTCCGAGGTTG 6FAM-CAGGGCGGCGGTCGAGCTC-BHQ1	45,206–45,226 45,268–45,249 45,247–45,229	AF268039	Mueller et al., 2002 [7]
PLHV-1 (1125) fw PLHV-1 rev PLHV-1 probe	CTC ACC TCC AAA TAC AGC GA GCT TGA ATC GTG TGT TCC ATA G 6FAM-CTG GTC TAC TGA ATC GCC GCT AAC AG-TAMRA	14,808–14,827 14,880–14,859 14,829–14,854	AF478169	Chmielewicz et al., 2003 [5]
PLHV-2 (1155) fw PLHV-2 rev PLHV-2 probe	GTC ACC TGC AAA TAC ACA GG GGC TTG AAT CGT ATG TTC CAT AT 6FAM-CTG GTC TAC TGA AGC GCT GCC AAT AG-TAMRA	10,814–10,833 10,887–10,865 10,835–10,860	AY170314	Chmielewicz et al., 2003 [5]
PLHV-3 /210 s) fw PLHV-3 (210as) rev PLHV-3 (210) probe	AAC AGC GCC AGA AAA AAA GG GGA AAG GTA GAA GGT GAA CCA TAA AA 6-FAM CCA AAG AGG AAA ATC-MGB	11,999–12,018 12,064–12,039 12,022–12,036	AY170315.1	McMahon et al., 2006 [25]
PRE-1 fwd PRE-1 rev PRE-1 probe	GACTAGGAACCATGAGGTTGCG AGCCTACACCACAGCCACAG FAM-TTTGATCCCTGGCCTTGCTCAGTGG-BHQ1	37–58 61–85 151–170	Y00104	Walker et al., 2003 [65]

## Data Availability

Data is contained within the article.

## Abbreviations

The following abbreviations are used in this manuscript:

AlHV-1	Alcelaphine herpesvirus type 1
DPS	Dippity pig syndrome
EBV	Epstein–Barr virus
HHV-4, HHV-8	Human herpesvirus 4, 8
IE	Immediate-early genes
KSHV	Kaposi sarcoma-associated herpesvirus
NF-κB	Nuclear factor κB
NFAT	Nuclear factor of activated T cells
OvHV-2	Ovine herpesvirus type 2
PBMCs	Peripheral blood mononuclear cells
PCMV/PRV	Porcine cytomegalovirus, a porcine roseolovirus virus
PLHV-1, PLHV-2, PLHV-3	Porcine lymphotropic herpesviruses -1, -2, and -3
SINE SuHV-1, SuHV-2, SuHV-3, SuHV-4, SuHV-5	Short Interspersed Nuclear Elements Suid herpesviruses 1, 2, 3, 4, and 5
vGPCRs	Viral G protein-coupled receptors

## References

[1] Ali A., Kemter E., Wolf E. (2024). Advances in Organ and Tissue Xenotransplantation. Annu. Rev. Anim. Biosci..

[2] Fishman J.A., Mueller N.J. (2024). Infectious Diseases and Clinical Xenotransplantation. Emerg. Infect. Dis..

[3] Griffith B.P., Goerlich C.E., Singh A.K., Rothblatt M., Lau C.L., Shah A., Lorber M., Grazioli A., Saharia K.K., Hong S.N. (2022). Genetically Modified Porcine-to-Human Cardiac Xenotransplantation. N. Engl. J. Med..

[4] Ehlers B., Ulrich S., Goltz M. (1999). Detection of Two Novel Porcine Herpesviruses with High Similarity to Gammaherpesviruses. J. Gen. Virol..

[5] Chmielewicz B., Goltz M., Franz T., Bauer C., Brema S., Ellerbrok H., Beckmann S., Rziha H.J., Lahrmann K.H., Romero C. (2003). A Novel Porcine Gammaherpesvirus. Virology.

[6] Denner J. (2021). Porcine Lymphotropic Herpesviruses (PLHVs) and Xenotransplantation. Viruses.

[7] Mueller N.J., Barth R.N., Yamamoto S., Kitamura H., Patience C., Yamada K., Cooper D.K., Sachs D.H., Kaur A., Fishman J.A. (2002). Activation of Cytomegalovirus in Pig-to-Primate Organ Xenotransplantation. J. Virol..

[8] Tucker A., McNeill F., Meehan B., Galbraith D., McArdle P., Allan G., Patience C. (2003). Methods for the Exclusion of Circoviruses and Gammaherpesviruses from Pigs. Xenotransplantation.

[9] Dor F.J., Doucette K.E., Mueller N.J., Wilkinson R.A., Bajwa J.A., McMorrow I.M., Tseng Y.L., Kuwaki K., Houser S.L., Fishman J.A. (2004). Posttransplant Lymphoproliferative Disease after Allogeneic Transplantation of the Spleen in Miniature Swine. Transplantation.

[10] Doucette K., Dor F.J., Wilkinson R.A., Martin S.I., Huang C.A., Cooper D.K., Sachs D.H., Fishman J.A. (2007). Gene Expression of Porcine Lymphotropic Herpesvirus-1 in Miniature Swine with Posttransplant Lymphoproliferative Disorder. Transplantation.

[11] Huang C., Fuchimoto Y., Gleit Z., Ericsson T., Griesemer A., Scheier-Dolberg R., Melendy E., Kitamura H., Fishman J., Ferry J. (2001). Posttransplantation Lymphoproliferative Disease in Miniature Swine after Allogeneic Hematopoietic Cell Transplantation: Similarity to Human PTLD and Association with a Porcine Gammaherpesvirus. Blood.

[12] Morozov V.A., Plotzki E., Rotem A., Barkai U., Denner J. (2016). Extended Microbiological Characterization of Göttingen Minipigs: Porcine Cytomegalovirus and Other Viruses. Xenotransplantation.

[13] Plotzki E., Keller M., Ehlers B., Denner J. (2016). Immunological Methods for the Detection of Porcine Lymphotropic Herpesviruses (PLHV). J. Virol. Methods.

[14] Krüger L., Kristiansen Y., Reuber E., Möller L., Laue M., Reimer C., Denner J. (2019). A Comprehensive Strategy for Screening for Xenotransplantation-Relevant Viruses in a Second Isolated Population of Göttingen Minipigs. Viruses.

[15] Jhelum H., Grand N., Jacobsen K.R., Halecker S., Salerno M., Prate R., Krüger L., Kristiansen Y., Krabben L., Möller L. (2023). First Virological and Pathological Study of Göttingen Minipigs with Dippity Pig Syndrome (DPS). PLoS ONE.

[16] Plotzki E., Heinrichs G., Kubícková B., Ulrich R.G., Denner J. (2016). Microbiological Characterization of a Newly Established Pig Breed, Aachen Minipigs. Xenotransplantation.

[17] Halecker S., Metzger J., Strube C., Krabben L., Kaufer B., Denner J. (2021). Virological and Parasitological Characterization of Mini-LEWE Minipigs Using Improved Screening Methods and an Overview of Data on Various Minipig Breeds. Microorganisms.

[18] Jhelum H., Papatsiros V., Papakonstantinou G., Krabben L., Kaufer B., Denner J. (2024). Screening for Viruses in Indigenous Greek Black Pigs. Microorganisms.

[19] Halecker S., Papatsiros V., Psalla D., Krabben L., Kaufer B., Denner J. (2022). Virological Characterization of Pigs with Erythema Multiforme. Microorganisms.

[20] Jhelum H., Kaufer B., Denner J. (2024). Application of Methods Detecting Xenotransplantation-Relevant Viruses for Screening German Slaughterhouse Pigs. Viruses.

[21] Mueller N.J., Livingston C., Knosalla C., Barth R.N., Yamamoto S., Gollackner B., Dor F.J., Buhler L., Sachs D.H., Yamada K. (2004). Activation of Porcine Cytomegalovirus, but Not Porcine Lymphotropic Herpesvirus, in Pig-to-Baboon Xenotransplantation. J. Infect. Dis..

[22] Cybulski P., Socha W., Jabłoński A., Kondratiuk R., Rybkowska W., Stadejek T., Larska M. (2025). First Molecular Detection of Porcine Cytomegalovirus (PCMV) and Porcine Lymphotropic Herpesvirus (PLHV) in Domestic Pigs in Poland. Pathogens.

[23] Dall Agnol A.M., Leme R.A., Suphoronski S.A., Oliveira T.E.S., Possatti F., Saporiti V., Headley S.A., Alfieri A.A., Alfieri A.F. (2020). Porcine lymphotropic herpesvirus DNA detection in multiple organs of pigs in Brazil. Braz. J. Microbiol..

[24] Franzo G., Drigo M., Legnardi M., Grassi L., Menandro M.L., Pasotto D., Cecchinato M., Tucciarone C.M. (2021). Porcine Gammaherpesviruses in Italian Commercial Swine Population: Frequent but Harmless. Pathogens.

[25] McMahon K.J., Minihan D., Campion E.M., Loughran S.T., Allan G., McNeilly F., Walls D. (2006). Infection of pigs in Ireland with lymphotropic gamma-herpesviruses and relationship to postweaning multisystemic wasting syndrome. Vet. Microbiol..

[26] Porto G.S., Leme R.A., Dall Agnol A.M., de Souza T.C.G.D., Alfieri A.A., Alfieri A.F. (2021). Porcine lymphotropic herpesvirus (Gammaherpesvirinae) DNA in free-living wild boars (*Sus scrofa* Linnaeus, 1758) in Brazil. J. Vet. Sci..

[27] Brema S., Lindner I., Goltz M., Ehlers B. (2008). Development of a recombinant antigen-based ELISA for the sero-detection of porcine lymphotropic herpesviruses. Xenotransplantation.

[28] Halecker S., Hansen S., Krabben L., Ebner F., Kaufer B., Denner J. (2022). How, where and when to screen for porcine cytomegalovirus (PCMV) in donor pigs for xenotransplantation. Sci. Rep..

[29] Russell G.C., Stewart J.P., Haig D.M. (2009). Malignant catarrhal fever: A review. Vet. J..

[30] Längin M., Mayr T., Reichart B., Michel S., Buchholz S., Guethoff S., Dashkevich A., Baehr A., Egerer S., Bauer A. (2018). Consistent success in life-supporting porcine cardiac xenotransplantation. Nature.

[31] Denner J., Bigley T.M., Phan T.L., Zimmermann C., Zhou X., Kaufer B.B. (2019). Comparative Analysis of Roseoloviruses in Humans, Pigs, Mice, and Other Species. Viruses.

[32] Denner J., Längin M., Reichart B., Krüger L., Fiebig U., Mokelke M., Radan J., Mayr T., Milusev A., Luther F. (2020). Impact of porcine cytomegalovirus on long-term orthotopic cardiac xenotransplant survival. Sci. Rep..

[33] Krüger L., Böttger J., Huang C.A., Denner J. (2021). Absence of porcine endogenous retrovirus (PERV) production from pig lymphoma cell lines. Virus Res..

[34] Jhelum H., Bender M., Reichart B., Mokelke M., Radan J., Neumann E., Krabben L., Abicht J.M., Kaufer B., Längin M. (2023). Evidence for Microchimerism in Baboon Recipients of Pig Hearts. Viruses.

[35] Fiebig U., Abicht J.M., Mayr T., Längin M., Bähr A., Guethoff S., Falkenau A., Wolf E., Reichart B., Shibahara T. (2018). Distribution of Porcine Cytomegalovirus in Infected Donor Pigs and in Baboon Recipients of Pig Heart Transplantation. Viruses.

[36] Yamada K., Tasaki M., Sekijima M., Wilkinson R.A., Villani V., Moran S.G., Cormack T.A., Hanekamp I.M., Hawley R.J., Arn J.S. (2014). Porcine cytomegalovirus infection is associated with early rejection of kidney grafts in a pig to baboon xenotransplantation model. Transplantation.

[37] Sekijima M., Waki S., Sahara H., Tasaki M., Wilkinson R.A., Villani V., Shimatsu Y., Nakano K., Matsunari H., Nagashima H. (2014). Results of Life-Supporting Galactosyltransferase Knockout Kidneys in Cynomolgus Monkeys Using Two Different Sources of Galactosyltransferase Knockout Swine. Transplantation.

[38] Denner J. (2018). Reduction of the survival time of pig xenotransplants by porcine cytomegalovirus. Virol. J..

[39] Mohiuddin M.M., Singh A.K., Scobie L., Goerlich C.E., Grazioli A., Saharia K., Crossan C., Burke A., Drachenberg C., Oguz C. (2023). Graft dysfunction in compassionate use of genetically engineered pig-to-human cardiac xenotransplantation: A case report. Lancet.

[40] Morozov V.A., Abicht J.M., Reichart B., Mayr T., Guethoff S., Denner J. (2016). Active replication of porcine cytomegalovirus (PCMV) following transplantation of a pig heart into a baboon despite undetected virus in the donor pig. Ann. Virol. Res..

[41] Denner J. (2025). How Does a Porcine Herpesvirus, PCMV/PRV, Induce a Xenozoonosis. Int. J. Mol. Sci..

[42] Issa N.C., Wilkinson R.A., Griesemer A., Cooper D.K., Yamada K., Sachs D.H., Fishman J.A. (2008). Absence of replication of porcine endogenous retrovirus and porcine lymphotropic herpesvirus type 1 with prolonged pig cell microchimerism after pig-to-baboon xenotransplantation. J. Virol..

[43] Mueller N.J., Kuwaki K., Knosalla C., Dor F.J., Gollackner B., Wilkinson R.A., Arn S., Sachs D.H., Cooper D.K., Fishman J.A. (2005). Early weaning of piglets fails to exclude porcine lymphotropic herpesvirus. Xenotransplantation.

[44] Egerer S., Fiebig U., Kessler B., Zakhartchenko V., Kurome M., Reichart B., Kupatt C., Klymiuk N., Wolf E., Denner J. (2018). Early weaning completely eliminates porcine cytomegalovirus from a newly established pig donor facility for xenotransplantation. Xenotransplantation.

[45] Goltz M., Ericsson T., Patience C., Huang C.A., Noack S., Sachs D.H., Ehlers B. (2002). Sequence analysis of the genome of porcine lymphotropic herpesvirus 1 and gene expression during posttransplant lymphoproliferative disease of pigs. Virology.

[46] Rovnak J., Quackenbush S.L., Reyes R.A., Baines J.D., Parrish C.R., Casey J.W. (1998). Detection of a novel bovine lymphotropic herpesvirus. J. Virol..

[47] Li H., Keller J., Knowles D.P., Crawford T.B. (2001). Recognition of another member of the malignant catarrhal fever virus group: An endemic gammaherpesvirus in domestic goats. J. Gen. Virol..

[48] Li H., Keller J., Knowles D.P., Taus N.S., Oaks J.L., Crawford T.B. (2005). Transmission of caprine herpesvirus 2 in domestic goats. Vet. Microbiol..

[49] Zhu H., Huang Q., Hu X., Chu W., Zhang J., Jiang L., Yu X., Zhang X., Cheng S. (2018). Caprine herpesvirus 2-associated malignant catarrhal fever of captive sika deer (Cervus nippon) in an intensive management system. BMC Vet. Res..

[50] Ulrich S., Goltz M., Ehlers B. (1999). Characterization of the DNA Polymerase Loci of the Novel Porcine Lymphotropic Herpesviruses 1 and 2 in Domestic and Feral Pigs. J. Gen. Virol..

[51] Allen U., Preiksaitis J., AST Infectious Diseases Community of Practice (2009). Epstein-Barr virus and posttransplant lymphoproliferative disorder in solid organ transplant recipients. Am. J. Transplant..

[52] El-Mallawany N.K., Rouce R.H. (2024). EBV and post-transplant lymphoproliferative disorder: A complex relationship. Hematol. Am. Soc. Hematol. Educ. Program.

[53] Lindner I., Ehlers B., Noack S., Dural G., Yasmum N., Bauer C., Goltz M. (2007). The porcine lymphotropic herpesvirus 1 encodes functional regulators of gene expression. Virology.

[54] Zuo J., Currin A., Griffin B.D., Shannon-Lowe C., Thomas W.A., Ressing M.E., Wiertz E.J.H.J., Rowe M., Früh K. (2009). The Epstein-Barr Virus G-Protein-Coupled Receptor Contributes to Immune Evasion by Targeting MHC Class I Molecules for Degradation. PLoS Pathog..

[55] Zuo J., Quinn L.L., Tamblyn J., Thomas W.A., Feederle R., Delecluse H.J., Hislop A.D., Rowe M. (2011). The Epstein-Barr Virus-Encoded BILF1 Protein Modulates Immune Recognition of Endogenously Processed Antigen by Targeting Major Histocompatibility Complex Class I Molecules Trafficking on Both the Exocytic and Endocytic Pathways. J. Virol..

[56] Griffin B.D., Gram A.M., Mulder A., Van Leeuwen D., Claas F.H., Wang F., Ressing M.E., Wiertz E. (2013). EBV BILF1 Evolved to Downregulate Cell Surface Display of a Wide Range of HLA Class I Molecules Through Their Cytoplasmic Tail. J. Immunol..

[57] Quinn L.L., Zuo J., Abbott R.J., Shannon-Lowe C., Tierney R.J., Hislop A.D., Rowe M., Rooney C.M. (2014). Cooperation Between Epstein-Barr Virus Immune Evasion Proteins Spreads Protection From CD8⁺ T Cell Recognition Across All Three Phases of the Lytic Cycle. PLoS Pathog..

[58] Paulsen S.J., Rosenkilde M.M., Eugen-Olsen J., Kledal T.N. (2005). Epstein-Barr Virus-Encoded BILF1 is a Constitutively Active G Protein-Coupled Receptor. J. Virol..

[59] Lyngaa R., Norregaard K., Kristensen M., Kubale V., Rosenkilde M.M., Kledal T.N. (2010). Cell Transformation Mediated by the Epstein-Barr Virus G Protein-Coupled Receptor BILF1 is Dependent on Constitutive Signaling. Oncogene.

[60] Mavri M., Kubale V., Depledge D.P., Zuo J., Huang C.A., Breuer J., Vrecl M., Jarvis M.A., Jovičić E.J., Petan T. (2022). Epstein-Barr Virus-Encoded BILF1 Orthologues from Porcine Lymphotropic Herpesviruses Display Common Molecular Functionality. Front. Endocrinol..

[61] Santoni F., Lindner I., Caselli E., Goltz M., Di Luca D., Ehlers B. (2006). Molecular interactions between porcine and human gammaherpesviruses: Implications for xenografts?. Xenotransplantation.

[62] Ehlers B. (2002).

[63] Duvigneau J., Hartl R., Groiss S., Gemeiner M. (2005). Quantitative simultaneous multiplex real-time PCR for the detection of porcine cytokines. J. Immunol. Methods.

[64] Behrendt R., Fiebig U., Norley S., Gürtler L., Kurth R., Denner J. (2009). A Neutralization Assay for HIV-2 Based on Measurement of Provirus Integration by Duplex Real-Time PCR. J. Virol. Methods.

[65] Walker J.A., Hughes D.A., Anders B.A., Shewale J., Sinha S.K., Batzer M.A. (2003). Quantitative intra-short interspersed element PCR for species-specific DNA identification. Anal. Biochem..

